# Neural responses to social touch with different emotional valences: an fNIRS study

**DOI:** 10.1093/scan/nsaf066

**Published:** 2025-06-30

**Authors:** Zhe Tang, Qin Luo, Yixian Huang, Shuo Zhao

**Affiliations:** School of Psychology, Shenzhen University, Shenzhen, 518060, China; School of Psychology, Shenzhen University, Shenzhen, 518060, China; School of Psychology, Shenzhen University, Shenzhen, 518060, China; School of Psychology, Shenzhen University, Shenzhen, 518060, China; Shenzhen Key Lab Affect & Social Neuroscience, Shenzhen University, Shenzhen, 518060, China; Key Laboratory of Brain Cognition and Emotional Health of Guangdong Higher Education Institutes, Shenzhen University, Guangdong, China

**Keywords:** social touch, C-tactile afferents, fNIRS, the prefrontal cortex, emotional valence

## Abstract

Social touch conveys a wealth of social and emotional information in interpersonal contexts. Despite the complexity of social touch in real-life situations, past research has often focused on CT-targeted social touch, neglecting the social-emotional expression in real social touch. Thus, we created a social touch paradigm in which the stimuli were derived from the Social Affective Touch Database. Using fNIRS, we investigated neural responses to social touch under different emotional valences. Our study found that under different touch conditions, the primary somatosensory cortex (S1), the superior temporal sulcus (STS), and the prefrontal cortex (PFC) were broadly activated. Additionally, we also found that the PFC mediates the pathway between S1 and Premotor Cortex-Somatomotor Cortex (PMC-SMC) for different emotional valence stimuli in the tactile modality. Our findings contribute to a deeper understanding of how social touch is linked to social emotions of different valences and ultimately heightened our awareness of the PFC function in social cognition and emotional processing.

## Introduction

Social touch plays a crucial role in the process of human socialization by conveying affective significance and sustaining social bonds (McGlone et al. 2014, Shamloo et al. 2020). As the most primal form of sensory experience, touch not only transmits information about the internal environment but also serves as an essential agent for successful social interaction (Gallace and Spence 2010). The function of the skin transcends mere tactile sensation, evolving into an organ that integrates social factors (Morrison et al. 2010). Indeed, handshakes, hugs, kisses, and other forms of social contact awaken pleasant tactile experiences and enhance intimacy (von Mohr et al. 2017, Packheiser et al. 2022). However, unpleasant forms of social contact such as hitting, restraining, and shaking evoke painful emotional experiences and disrupt the relationship between the initiator and the recipient of the touch (Peled-Avron et al. 2016, Hall et al. 2019).

Over the past decade, psychologists and neuroscientists have shown considerable interest in exploring the influence of touch within social contexts, such as the transmission of emotions through touch (Hertenstein et al. 2009, Kirsch et al. 2018), the neural mechanisms of touch (Gordon et al. 2013, Lopez-Sola et al. 2019), and the factors affecting touch perception (Klöcker et al. 2013, Suvilehto et al. 2023). Research on touch helps deepen our understanding of social behavior and improves interventions in clinical treatment. Specifically, different social settings (public vs. private) create diverse forms of tactile interactions, with most intimate physical contact occurring in private settings (Willis and Briggs 1992). Emotional meanings conveyed during tactile interactions also differ between family members and strangers: strangers convey universal and prosocial emotions via touch whereas family members are found to express social control and a wider range of affective states, even including fear and envy (Thompson and Hampton 2011, Suvilehto et al. 2015). However, when cognitive neuroscientists attempt to furthermore explore the neural mechanisms influenced by touch within social contexts, a key question is how to define the manifestation of touch in both natural and scientific contexts.

To address this question, some neuroscientists have developed an operationally defined social touch stimulus (i.e. touch at 3 cm/s targeting CT fibers on the skin, also defined as “CT-touch”) to elucidate the neural substrates of social touch (McGlone et al. 2014, Cascio et al. 2019, Schirmer et al. 2023). These findings indicates that social touch, as represented by CT-touch, activates specific cortical regions, including the primary somatosensory cortex (S1), superior temporal sulcus (STS), insula, medial prefrontal cortex (mPFC), orbitofrontal cortex (OFC), and dorsal anterior cingulate cortex (dACC) (Lindgren et al. 2012, Voos et al. 2013). Notably, there is consistent activation observed in the temporal cortex, parietal cortex, and frontal cortex during social touch. Specifically, the STS, a critical node of the social brain, shows significant activation in both children and adults in response to CT-touch (Bjornsdotter et al. 2014, Davidovic et al. 2016). The parietal cortex also plays a role in the processing of social touch. Studies have shown that the S1 is involved in the affective dimension of somatosensation (Gazzola et al. 2012). From the perspective of the sensory qualities of social touch, CT-touch indeed establishes the neural basis of social touch.

However, as more researchers focus on the naturalness and ecological validity of experimental studies, the second definition of social touch (i.e. the interpersonal and intentional aspects: whether the touch conveys emotional signals) has gradually gained favor (Gliga et al. 2019). The CT-touch emphasizes specific ways of touching certain body parts, such as using a brush to stroke the forearm (Bennett et al. 2014, PirazzoLi et al. 2019) or using feathers to stroke the leg (Mayorova et al. 2023). The naturalistic and ecological definitions focus on different types of touch (e.g. light tapping, hugging, handshakes, and hitting) that convey information about the toucher’s feelings and intentions towards the recipient. More importantly, the experimental paradigm that standardizes social touch based on CT-targeted touch overlooks the naturalistic aspects of social interactions while overemphasizing the hedonic properties inherent to CT touch (Anderson 1978, Field 2010, Gallace and Spence 2010, Packheiser et al. 2024). This approach neglects a comprehensive exploration of the emotional valence dimensions of social touch. Consequently, the complex, diverse, and multilayered nature of social touch expressions should not be reduced to a single neural system or afferent pathway (Suvilehto et al. 2023). It is essential to investigate the neural mechanisms underlying naturalistic social touch to capture its full complexity and richness. To our knowledge, complex social touch has primarily been explored in studies using static images (Peled-Avron et al. 2016, Schirmer et al. 2023), and has not yet been investigated with dynamic real human social touch simulation.

Based on the emotional information conveyed by different forms of social touch, these were categorized into two opposing dimensions according to the valence model (pleasant—unpleasant) ­(Russell 1980). Social touch, represented by CT-touch, predominantly conveys pleasant and positive emotional signals, while there is a lack of research on the neural mechanisms of unpleasant and negative social touch. To the best of our knowledge, there is limited research on the neural mechanisms of unpleasant but harmless social touch. A study using EEG indicated that social touch reduced frontal theta power during negative emotional imagery (Kraus et al. 2020). In fMRI studies, elastic bands were used to elicit a unpleasant touch experience. The results indicate that receiving unpleasant touch triggers stronger activation in the somatosensory cortex, insula, and cingulate cortex (Masson and Isik 2021). However, neither has examined unpleasant touch from the perspective of social and interpersonal emotional intentions, leaving room for exploration of the neural mechanisms underlying pleasant and unpleasant touch in real-life situations.

Given the role of the PFC in emotion comprehension and regulation, the cortical connections related to the different valence dimensions of social touch are particularly noteworthy (Gyurak et al. 2011, Dixon et al. 2017). For instance, fMRI studies on different valences within the visual modality have revealed the hemodynamic mediation role of the PFC. Specifically, the ventrolateral prefrontal cortex (VLPFC) mediates the integration of emotion processing in response to visually induced emotional valences (Hoshi et al. 2011, Kida and Hoshi 2016). A recent study using fNIRS has provided direct evidence supporting the involvement of the PFC in tactile processing. Specifically, the PFC was found to play a crucial role in evaluating the emotional valence and showed strong neural correlates with the perceived intensity (Abagnale et al. 2025). Similarly, mediation has been observed in the tactile modality, where changes in the oxyhemoglobin (Oxy-Hb) pathway between S1 and the PFC, under soft touch stimuli primarily involving massage, are mediated by the STS, despite this touch lacking specific affective in nature (Li et al. 2019). However, these studies have not systematically examined the brain region changes induced by real social touch across different valences.

This fNIRS study aimed to identify the brain responses elicited by social touch in naturalistic settings and to provide new insights for future research on the cortical mechanisms underlying real-world social touch. Accordingly, we hypothesize that neural responses to social touch tasks in a natural environment, across different valence dimensions, involve brain regions associated with interoceptive processing, social cognition, and emotion regulation. Additionally, to further explore and elucidate the relationships between cortical areas during social touch with varying valence, particularly in identifying the direct or indirect roles of brain regions along the valence dimension, this study also hypothesizes that the PFC mediates the effects of social touch across different valences.

## Materials and methods

### Participants

We have estimated the sample size using a prior power analysis conducted through G*Power (version 3.1.9.7). For a two-tailed bivariate paired samples *t*-test with a medium effect size of 0.5, 34 participants could achieve 80% statistical power. Eventually, forty male adults were recruited for this study. Similar to many prior studies, the sample size in this study is entirely sufficient (Ellingsen et al. 2014, Panagiotopoulou et al. 2017, Bershad et al. 2019). Previous investigations have used a combination of males and females (Trotter et al. 2016, Yu et al. 2019, Wijaya et al. 2020). However, the emphasis has primarily been on CT-touch triggered by objects rather than skin-to-skin touch. Therefore, we recruited only males in the current study to minimize the variability of our sample and mitigate the confounding effects of gender interactions that have proven to cause social desirability (Cheng et al. 2015, Baker et al. 2016). As this study is a preliminary exploratory investigation, we plan to specifically address the important issue of gender pairings in brain synchrony mechanisms to social touch in future research. Eight participants were excluded from further data analysis due to low-quality fNIRS data (*n* = 2) or to technical issues (*n* = 6). The final sample comprised 32 participants, aged between 18 and 26 years (M = 21.44; SD = 2.41). All were right-handed and had no history of psychiatric or neurological diagnoses or tactile impairments. All participants were naive to the experimental purpose and provided written informed consent before participation. Participants received compensation for their involvement. The study obtained approval from the Medical Ethical Committee of Shenzhen University.

### Experimental design

#### Social touch paradigm

**Figure 1.**
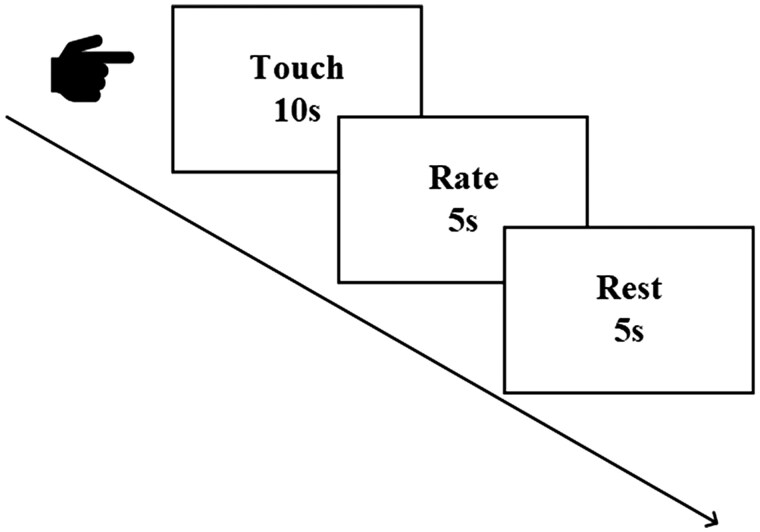
Experimental Design: Social touch paradigm: Pleasant touch or Unpleasant touch, lasting for 10 s; Rating tasks: Pleasantness ratings and Arousal ratings (1–9 point), lasting for 5 s; Rest period lasts 5 s.

In line with the procedures of previous social touch experiments (Gazzola et al. 2012, Lucas et al. 2015), participants were informed that they would receive touch from either a male or female experimenter during the formal task in the current study. However, they actually were only touched by a male experimenter to minimize variability introduced by gender interactions. Additionally, the male and female experimenters were dressed similarly, with comparable coverage of their bodies and arms, to prevent participants from guessing the gender of the toucher. Throughout the experiment, participants wore blindfolds and remained still to focus on the tactile sensations, eliminating visual stimuli across all conditions. The experimental design involved randomized stimuli (see Fig. 1), each lasting 10 s and comprising two categories of social touch stimuli, each with eight different manifestations (eight types of positive stimuli: handshake, hug, shoulder massage, a close touch of upper arm, distant touch of upper arm, neck caress, hands on shoulders from behind, comforting hug with patting; eight types of negative stimuli: back slap, shoulder slap, forearm slap, neck slap from behind, shoulder shake, forceful shoulder tap from behind, forceful elbow grab, hand restraint. For detailed evaluations of social touch stimuli, see Supplementary Appendix 2). These stimuli represented common types of social touch encountered in daily life and were previously shown to evoke emotional experiences in touched individuals (Lee Masson and Op de Beeck 2018). Following each tactile task, there is a 5-s rating task, where participants rate the pleasantness (“How pleasant is the touch?”; 1-very unpleasant, 5-neutral, 9-very pleasant) and arousal (“How arousing is the touch?”; 1-very calm, 5-neutral, 9-very exciting) of tactile stimuli on a 9-point Likert scale. After the ratings, participants have a 5-s rest period until the next stimulation begins. The entire experiment lasts 5.53 minutes.

### fNIRS assessment

#### NIRS probe

**Figure 2.**
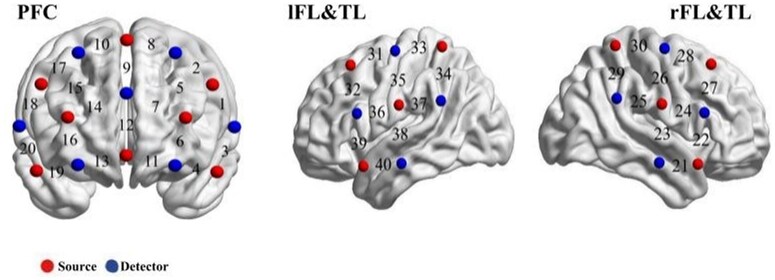
Probe configuration: Numerals on the cerebral cortex represent the recording channels (CHs). The red dots are the sources and the blue dots are the detectors. PFC: the prefrontal cortex, r/lFL&TL: the right/left frontal lobe & temporal lobe.

Brain activity was recorded using two NIRSport continuous-wave fNIRS devices (NIRx Medical Technologies, LLC, USA) operating at in tandem mode within the NIRStar acquisition software (version 15.3), with a sampling rate of 3.47 Hz. The montage of the optodes was established utilizing NIRSite 2.0 (NIRx Medical Technologies, LLC). Sixteen LED sources that emitted light at wavelengths of 760 and 850 nm and 16 avalanche photodiode detectors were aligned, each separated by an inter-optode distance of approximately 3 cm. Two configurations of optodes were applied in the present study. The first configuration consisted of eight sources and seven detectors, forming 20 channels, covering the PFC. The second configuration was placed separately on the right and left hemispheres, with four sources and four detectors in each hemisphere, forming 10 channels, covering the frontal lobe and temporal lobe (FL&TL). The source and detector were placed according to the 10-10 system, with PO4 as references (see Fig. 2). Lastly, the locations of 40 channels were transcribed the Montreal Neurological Institute and Hospital (MNI) coordinates, and the percentage of Brodmann areas was calculated using the NIRS-SPM toolbox (Ye et al. 2009). The specific locations of each channel are detailed in Supplementary Appendix 2.

### NIRS data analysis

Data from fNIRS optodes was analyzed using NIRS-SPM version 4.1 (Ye et al. 2009, Tak et al. 2011) implemented in MATLAB (Mathworks, Sherborn, MA, USA), with non-specific global trends due to breathing, cardiac, vaso-motion or other experimental errors being effectively removed by the Wavelet-minimum description length (MDL) detrending algorithm. Additionally, this study employs the Precoloring method (Worsley and Friston 1995) along with a low-pass filter of the hemodynamic response function (HRF) to mitigate random noise from instrumentation and physiological noise arising from heart rate and respiration. For general linear model (GLM) analyses, 10 s blocks of tactile stimulus and 5 s blocks of rest after each positive touch or negative touch were modeled separately with boxcar functions. We then convolved four task events (positive touch, negative touch, rest after positive touch, and rest after negative touch) with a hemodynamic response curve to model the hypothesized oxy-Hb response during each experimental condition. In line with many other studies β values used as indices of activation in the corresponding brain regions (Suzuki et al. 2008, Bennett et al. 2014, Byun et al. 2014, Muthalib et al. 2015, Li et al. 2019). Statistical analysis was conducted using SPSS 20.0 software (IBM, Somers, USA). Paired *t*-tests were performed on the β values for each channel under the two primary contrast conditions (positive touch > rest after positive touch, negative touch > rest after negative touch), with the significance level set at 0.05. Finally, the false discovery rate (FDR) method was applied to correct for multiple comparisons across channels, further reducing the false positive rate.

### Mediation analysis

The mediation analysis within the social affective touch paradigm involved averaging the trials for each experimental condition (pleasant touch and unpleasant touch) for each subject within a specified time window. The changes in oxy-hemoglobin (HbO_2_) concentration in activated channels were calculated under social affective touch stimuli. Data from 1 s before the stimulus onset served as the baseline, which was subtracted from the subsequent signal. The time window from 5 to 10 s post-stimulus was included in the analysis. Concentrations of HbO_2_ have been consistently shown to have a signal-to-noise ratio (SNR) and is more sensitive than HbR in reflecting task-induced, event-related cortical responses (Huppert et al. 2006, Jiang et al. 2015, Chen et al. 2023). Specifically, we employed the Bootstrap method to examine the mediation effect of the PFC or STS between S1 and Premotor Cortex-Somatomotor Cortex (PMC-SMC) (Preacher and Hayes 2008). This method has also been used to investigate the neural circuits involved in hand massage, thereby demonstrating that gentle human touch effectively activates the neural pathways related to social cognition and emotion (Li et al. 2019). In addition, correlation between HbO_2_ concentration in brain regions during social touch tasks and behavioral scores as well as questionnaire scores were calculated.

## Results

### Cortical activation in the social touch paradigm

**Figure 3.**
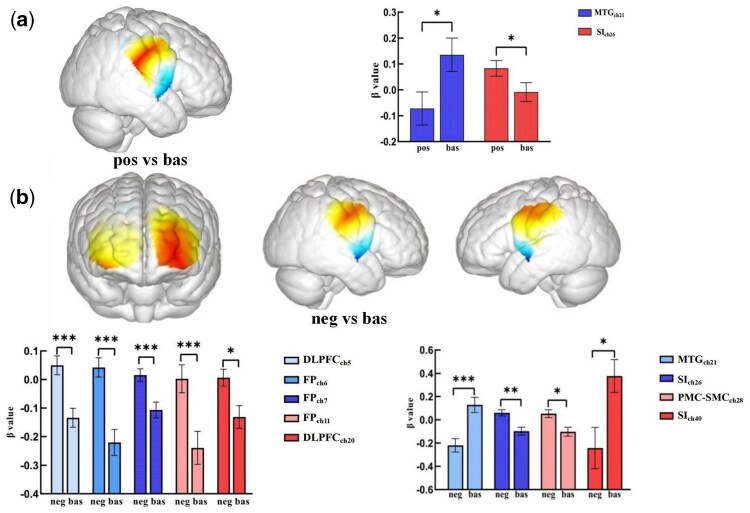
Activation maps for the touch task versus the baseline condition. The bar graphs display the β values (M±SEM) for: positive touch vs. baseline (a), and negative touch vs. baseline (b). The figure also shows the activation maps for the following comparisons: (1) Positive touch vs. baseline: activation in the MTG and the S1 (a). (2) Negative touch vs. baseline: activation in the DLPFC, the FP, the MTG, the S1, and the PMC—SMC (b). *P < .05, **P < .01, ***P < .001.

Results from the GLM analysis indicate that social-emotional touch stimuli of different valences elicit differential cortical activations. Firstly, compared to the resting condition, positive touch showed significant differences in channel 21 (*t *=* −*2.31, *P *= .03, *Cohen’s d *= 0.41) and channel 26 (*t *= 2.55, *P *= .02, *Cohen’s d *= 0.45). Meanwhile, negative touch showed significant differences in channel 5 (*t *= 3.77, *P *< .001, *Cohen’s d *= 0.67), channel 6 (*t *= 5.06, *P *< .001, *Cohen’s d *= 0.90), channel 7 (*t *= 3.83, *P *< .001, *Cohen’s d *= 0.68), channel 11 (*t *= 3.78, *P *< .001, *Cohen’s d *= 0.67), channel 20 (*t *= 2.88, *P *= .07, *Cohen’s d *= 0.51), channel 21 (*t *=* −*3.66, *P *< .001, *Cohen’s d *= 0.65), channel 26 (*t *= 3.15, *P *< .01, *Cohen’s d *= 0.56), channel 28 (*t *= 2.69, *P *= .01, *Cohen’s d *= 0.48), and channel 40 (*t *=* −*2.30, *P *< .05, *Cohen’s d *= 0.41) (see Fig. 3).

Based on the Brodmann area localization derived from the conversion of channel coordinates to MNI coordinates (see Supplementary Appendix 2) and the analysis results mentioned above, it is evident that pleasant touch elicited significant activation in the STS and S1 compared to the resting condition. However, unpleasant touch triggered significant activation in a broader range of brain regions, including the dorsolateral prefrontal cortex (DLPFC), the frontopolar area (FP), the STS, the S1, and the PMC-SMC.

### Mediation in the social touch paradigm

**Figure 4.**
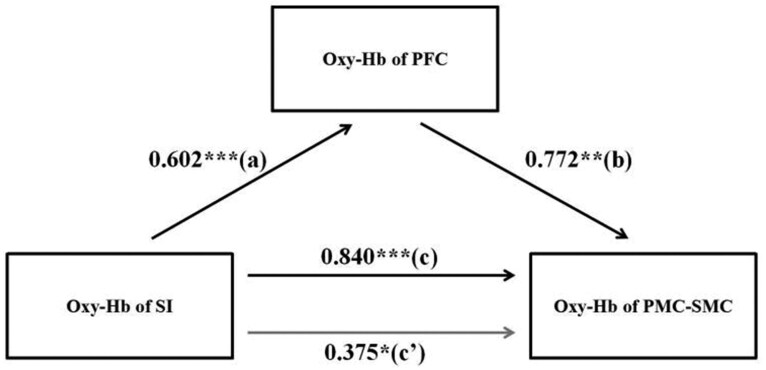
Mediation analysis between the three key brain regions in unpleasant touch. Mediation analysis between activities of SI-PFC and PMC-SMC (path a = 0.602, *P* < .001; path b = 0.772, *P* < .01; path c = 0.840, *P* < .001; path c’= 0.375, *P* = .048). Note: ***P < .001,**P < .01,*P < .05.

**Figure 5.**
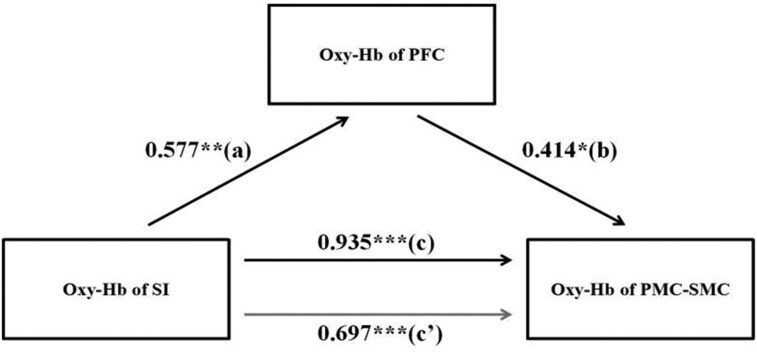
Mediation analysis between the three key brain regions in pleasant touch. Mediation analysis between activities of SI-PFC and PMC-SMC (path a = 0.577, *P* < .01; path b = 0.414, *P* < .05; path c = 0.935, *P* < .001; path c’ = 0.697, *P* ≤ .001). Note: ** *P* < .001, **P < .01, *P < .05.

To better understand whether the PFC mediates the effect of S1 on PMC-SMC, we further explored the relationships between cortical regions under different social affective tactile stimuli by calculating the HbO_2_ concentration during the social touch paradigm. Using the Bootstrap method (Preacher and Hayes 2008), we validated the mediating role of the PFC between S1 and PMC-SMC in the pleasant and unpleasant social touch. In the unpleasant social touch the changes in S1 were significantly associated with the changes in PFC (path a = 0.602) and the changes in PMC-SMC (path c = 0.840). When the changes in PFC were included as the predictor, they were significantly associated with the changes in PMC-SMC (path b = 0.772) and the correlation between the changes in S1 and PMC-SMC was attenuated (path c’ = 0.375). These results indicated partial mediation and using a bootstrap analysis the mediation was significant (indirect effect = 0.465, 95% CI = [0.033–0.766], bootstrap = 1000, see Fig. 4). Likewise, in the pleasant social touch the changes in S1 were significantly associated with the changes in PFC (path a = 0.577) and the changes in PMC-SMC (path c = 0.935). When the changes in PFC were included as the predictor, they were significantly associated with the changes in PMC-SMC (path b = 0.414) and the correlation between the changes in S1 and PMC-SMC was attenuated (path c’ = 0.697). These results indicated partial mediation and using a bootstrap analysis the mediation was significant (indirect effect = 0.239, 95% CI = [0.096–0.465], bootstrap = 1000, see Fig. 5). Both results therefore confirmed that the oxy-Hb changes in S1 mediated changes in PMC-SMC through increasing PFC activation rather than the STS.

## Discussion

In the present study, we aim to elucidate the neural response of social touch with different valences in naturalistic contexts, as well as the cortical connectivity involved in social touch under varying valences, particularly the mediating role of the PFC. Our findings support the first hypothesis: Social touch with different valences activates cortical responses, such as the S1, STS, DLPFC, and FP. These brain areas are closely associated with interoceptive processing, social cognition, and emotion regulation functions. Additionally, our further mediation analysis found that the PFC mediates the pathway between SI and PMC-SMC during social touch, regardless of valence. Our study lays the foundation for future exploration of the neural mechanisms underlying real-world social touch. Meanwhile, neuroimaging studies of social touch also contribute to explaining the biological mechanisms of the development of social emotions.

Our results revealed that specific cortical areas are activated during real social touch. These activated regions are similar to the CT-touch, including the S1, STS, and PFC (Lindgren et al. 2012, Gordon et al. 2013, Voos et al. 2013). Attentionally, the MTG exhibited negative activation under the positive touch. Similarly, temporal lobe regions associated with the social brain, such as the pSTS and MTG, also showed negative activation when adults received pleasant social touch (e.g. pressure massage) (Chen et al. 2023). This finding, to some extent, provides evidence supporting the strong activation of the frontal social brain, particularly the PFC, in response to naturalistic emotional social stimuli (Kida and Shinohara 2013, Abagnale et al. 2025). However, in contrast to studies directly observing social touch behaviour and its emotional significance in the cortex (Masson et al. 2018), real experiences derived from the social touch materials in the Social Affective Touch Database elicited broader activation in the PFC. Therefore, the cortical activation results under different valences of social touch strongly confirm that both the inherent physical properties and the socio-emotional attributes of social touch influence the neural responses during real touch (Gazzola et al. 2012, Cascio et al. 2019, Sailer and Leknes 2022).

Interestingly, when distinguishing the emotional valence of social touch, activation analysis results showed significant activation in the PMC-SMC during unpleasant but harmless social touch. This phenomenon may be due to the tight functional coupling between touch and movement, supported by neural connections between the somatosensory and motor systems. Tactile information, after initial processing in the primary somatosensory cortex (S1), is transmitted to the secondary somatosensory cortex (S2), posterior parietal cortex (PPC), premotor and supplementary motor cortex (PMC-SMC), and primary motor cortex (M1) (Delhaye et al. 2018). It is noteworthy that only unpleasant social touch activated the sensorimotor loop, unlike pleasant social touch. This may be partly because unpleasant social touch evokes a similar nociceptive network, including motor and premotor mechanisms related to pain avoidance responses (Valeriani et al. 1999, Garcia-Larrea and Peyron 2013). In the pain matrix, the somatosensory cortex is believed to play a role in the sensory discrimination of pain (Vierck et al. 2013). The anterior cingulate cortex (ACC), insular cortex, and amygdala are associated with affective-motivational components, while the PFC is related to cognitive evaluation (Bushnell et al. 2013, Veinante et al. 2013, Nakata et al. 2014). The PMC-SMC and M1 are involved in behavioral responses to nociceptive stimuli (Verriotis et al. 2016). Importantly, although touch and pain are closely related sensory modalities, distinguishing between social touch and pain is crucial, particularly in the neural mechanisms (Meijer et al. 2022). Negative social touch signals are transmitted via CT-fibers, activating emotional and social reward system such as the insula, ACC, OFC, and mPFC. In contrast, pain signals are innervated by A-delta and CT-fibers, engaging sensory-discriminative regions such as S1 and parietal operculum (Apkarian et al. 2005, Bourne et al. 2014, De Ridder et al. 2021), and motivational-affective regions such as anterior insula, ACC and amygdala (Starr et al. 2009, Neugebauer 2015, Isoda 2021). Therefore, the transmission through specialized nerve fibers drives both overlapping and distinct cortical activations for social touch and pain processing. To our knowledge, there is scant research on the neural mechanisms underlying the processing of unpleasant but non-harmful touch, as most studies focus on nociception and pain perception rather than unpleasant touch. Thus, it is essential for future research to distinguish whether the pain experience induced by unpleasant social touch is psychological or physiological in nature.

To investigate whether the PFC mediates cortical interactions under different valences of tactile modalities, we conducted a mediation analysis on oxy-Hb in the cortex during a social touch task. We found that the PFC serves as a mediator in both positive and negative valence conditions. Specifically, S1 activation during pleasant or unpleasant social touch primarily influences PMC-SMC activation via the PFC rather than directly. Therefore, pleasant and unpleasant social touch effectively activates specific brain circuits involved in social emotion regulation and sensorimotor functions. The mediation role of the PFC in both positive and negative valence further supports the involvement of PFC neural activity in encoding valence-related information, which is subsequently used to differentiate types of emotional processing (Chavez and Heatherton 2015). Furthermore, the brain regions mediated by this model are core sensorimotor areas, indicating that real social touch can trigger subconscious regulatory movements that influence dyadic interactions in social exchanges. There is ample neuroimaging evidence showing that humans mobilize two motor networks during social interactions: the direct motor network and the indirect motor network (Nutt et al. 2011, Zwergal et al. 2013, Herold et al. 2017). Notably, fMRI studies indicate that the indirect motor pathway is activated during imagined movement or movement induced by tactile stimuli, with neural commands transmitted through the PFC and the PMC-SMC to the basal ganglia, subthalamic nucleus, and midbrain motor areas (La Fougere et al. 2010, Limanowski et al. 2020, Boyne et al. 2021). Particularly, the mediating role of the STS was not replicated in the study, which may be attributed to differences in experimental paradigms (Trotter et al. 2016, Pawling et al. 2017) and the social selectivity of STS neural function (Beauchamp et al. 2004, Isik et al. 2017). Concurrently, the PFC’s mediating role further underscores its importance in affective-social reward processing, while the STS may not differentiate valence-specific responses. Based on these findings, we believe the construction of this model is justified, as it highlights the mediating role of emotional regulation in sensory perception and motor planning.

There are still some limitations in this study, which should be further explored in future studies. The primary limitation is that the decoding process of the meaning of social touch is influenced by various complex factors (e.g. culture, context, and expectations). While this study aimed to explore the neural responses to social touch with different valences in natural settings, future research should assess whether these neural characteristics are influenced by individual traits and contextual factors during touch. Additionally, although this study used fNIRS to optimize data collection during social touch, the penetration depth of near-infrared light into the brain (up to 2 cm from the skull) limits the exploration of deeper brain regions (e.g. the insula) (Golaszewski et al. 2006, Suvilehto et al. 2021). Finally, despite minimizing exclusion criteria in the experimental design, a few participants were not included in the final sample due to excessive motion artifacts or atypical emotional standards for tactile perception. Future research should aim to address these limitations with effective solutions.

This study contributes to expanding the understanding of brain responses to social touch in naturalistic contexts and offers a new perspective on the development of social emotions within the tactile modality. Exploring the impact of social touch with different valences on the cerebral cortex in a naturalistic setting addresses the limitations of previous research and provides a more comprehensive neural basis for understanding human social behavior. Moreover, the mediating role of the PFC in social touch with different valences not only deepens our understanding of PFC function but also broadens our comprehension of social emotions within sensory modalities. Importantly, the findings of this study offer guidance for future explorations of the neural mechanisms underlying social touch in real-world contexts, which will be instrumental in developing new clinical interventions, particularly for treating social disorders and autism spectrum disorder. In summary, this study provides ecologically valid cognitive neuroscience evidence that advances the theoretical understanding of social touch, while also offering behavioral and neural support for the development of social touch interventions.

## Conclusion

Overall, our study lays the foundation for future research on the neural characteristics of social touch in natural contexts. We successfully applied touch materials from a socio-emotional database to fNIRS research and identified cortical activation related to social touch of different valences. Interestingly, unpleasant social touch activated a network matrix similar to that associated with pain, which may indicate that the conveyance and perception of socio-emotional information through the tactile modality is closely related to its meaning (Wiech 2016). Additionally, the study demonstrated the mediating role of the PFC in emotional processing within the social touch, further emphasizing the PFC’s function in regulating emotional behavior. Exploring social touch with different valences helps to understand brain mechanisms from a socio-emotional perspective and provides new insights for clinical interventions in patients with social perception disorders.

## Supplementary Material

nsaf066_Supplementary_Data

## Data Availability

The datasets generated and/or analyzed during the current study are not publicly available due to privacy and ethical restrictions but are available from the corresponding author upon reasonable request.
