# Supplementary material for: Neural responses to social touch with different emotional valences: an fNIRS study
Source: Soc Cogn Affect Neurosci. 2025 Jun 30;20(1):nsaf066. doi: 10.1093/scan/nsaf066 (PMC12380468; doi:10.1093/scan/nsaf066)
Supplement: nsaf066_Supplementary_Data [file nsaf066_supplementary_data.zip › scan-24-277-File015.docx]

**Supplementary Materials**

No statistically significant correlations were found. For positive social touch, the correlations between STQ and significant channels were: *r_ch21_* = -0.04 and *r_ch26_* = -0.06 (all *ps* > 0.05). For negative social touch, the correlations were: *r_ch5_* = 0.15、*r_ch6_* = -0.15、*r_ch7_* = -0.04、*r_ch11_* = -0.09、*r_ch20_* = -0.11、*r_ch21_* = 0.15、*r_ch26_* = 0.07、*r_ch28_* = 0.18、*r_ch40_* = 0.23 (all *ps* > 0.05). See Appendix Table 3 for STQ descriptive statistics.

**Appendix Table 3: Descriptive Statistics of Scales (Mean ± SD)**

| Measurements（32 subjects） | Range | Mean ± SD |
| --- | --- | --- |
| Social Touch Questionnaire（STQ） | 16 - 55 | 36.34 ± 9.14 |
