# Supplementary material for: Neural responses to social touch with different emotional valences: an fNIRS study
Source: Soc Cogn Affect Neurosci. 2025 Jun 30;20(1):nsaf066. doi: 10.1093/scan/nsaf066 (PMC12380468; doi:10.1093/scan/nsaf066)
Supplement: nsaf066_Supplementary_Data [file nsaf066_supplementary_data.zip › scan-24-277-File013.docx]

| Channel | Optode  names | MNI coordinates  X Y Z | Brodmann area (BA) | Overlap |
| --- | --- | --- | --- | --- |
| CH1 | S1-D1 | -45 44 26 | 45 - pars triangularis Broca's area  46 - Dorsolateral prefrontal cortex | 0.71  0.29 |
| CH2 | S1-D2 | -29 45 43 | 9 - Dorsolateral prefrontal cortex  46 - Dorsolateral prefrontal cortex | 0.88  0.12 |
| CH3 | S2-D1 | -48 51 4 | 46 - Dorsolateral prefrontal cortex  45 - pars triangularis Broca's area | 0.85  0.15 |
| CH4 | S2-D3 | -34 65 -6 | 11 - Orbitofrontal area  10 - Frontopolar area  47 - Inferior prefrontal gyrus  46 - Dorsolateral prefrontal cortex | 0.42  0.38  0.13  0.07 |
| CH5 | S3-D2 | -23 57 36 | 9 - Dorsolateral prefrontal cortex  46 - Dorsolateral prefrontal cortex  10 - Frontopolar area | 0.54  0.38  0.08 |
| CH6 | S3-D3 | -24 70 8 | 10 - Frontopolar area  11 - Orbitofrontal area | 0.83  0.17 |
| CH7 | S3-D4 | -13 67 26 | 10 - Frontopolar area  9 - Dorsolateral prefrontal cortex | 0.94  0.06 |
| CH8 | S4-D2 | -10 46 52 | 9 - Dorsolateral prefrontal cortex  8 - Includes Frontal eye fields | 0.76  0.24 |
| CH9 | S4-D4 | 2 55 42 | 9 - Dorsolateral prefrontal cortex  10 - Frontopolar area | 0.97  0.03 |
| CH10 | S4-D5 | 13 46 53 | 9 - Dorsolateral prefrontal cortex  8 - Includes Frontal eye fields | 0.70  0.30 |
| CH11 | S5-D3 | -11 73 -2 | 10 - Frontopolar area  11 - Orbitofrontal area | 0.58  0.42 |
| CH12 | S5-D4 | 3 69 17 | 10 - Frontopolar area | 1 |
| CH13 | S5-D6 | 15 73 -2 | 10 - Frontopolar area  11 - Orbitofrontal area | 0.56  0.44 |
| CH14 | S6-D4 | 17 67 26 | 10 - Frontopolar area  9 - Dorsolateral prefrontal cortex | 0.95  0.05 |
| CH15 | S6-D5 | 27 56 36 | 9 - Dorsolateral prefrontal cortex  46 - Dorsolateral prefrontal cortex  10 - Frontopolar area | 0.56  0.40  0.04 |
| CH16 | S6-D6 | 30 68 8 | 10 - Frontopolar area  11 - Orbitofrontal area | 0.85  0.15 |
| CH17 | S7-D5 | 33 44 44 | 9 - Dorsolateral prefrontal cortex  46 - Dorsolateral prefrontal cortex | 0.94  0.06 |
| CH18 | S7-D7 | 49 42 27 | 45 - pars triangularis Broca's area  46 - Dorsolateral prefrontal cortex | 0.88  0.12 |
| CH19 | S8-D6 | 38 65 -6 | 10 - Frontopolar area  11 - Orbitofrontal area  47 - Inferior prefrontal gyrus  46 - Dorsolateral prefrontal cortex | 0.39  0.36  0.15  0.10 |
| CH20 | S8-D7 | 51 49 4 | 46 - Dorsolateral prefrontal cortex  45 - pars triangularis Broca's area | 0.79  0.21 |
| CH21 | S9-D9 | 70 -8 -8 | 21 - Middle Temporal gyrus  22 - Superior Temporal Gyrus | 0.93  0.07 |
| CH22 | S9-D10 | 65 9 10 | 6 - Pre-Motor and Supplementary Motor Cortex  48 - Retrosubicular area  44 - pars opercularis, part of Broca's area  43 - Subcentral area | 0.47  0.40  0.09  0.04 |
| CH23 | S10-D9 | 72 -19 11 | 22 - Superior Temporal Gyrus  21 - Middle Temporal gyrus  43 - Subcentral area | 0.89  0.10  0.01 |
| CH24 | S10-D10 | 68 -3 28 | 43 - Subcentral area  6 - Pre-Motor and Supplementary Motor Cortex  4 - Primary Motor Cortex | 0.81  0.12  0.07 |
| CH25 | S10-D11 | 70 -33 32 | 40 - Supramarginal gyrus part of Wernicke's area  2 - Primary Somatosensory Cortex  48 - Retrosubicular area | 0.50  0.37  0.13 |
| CH26 | S10-D12 | 64 -17 45 | 1 - Primary Somatosensory Cortex  3 - Primary Somatosensory Cortex  4 - Primary Motor Cortex  2 - Primary Somatosensory Cortex  43 - Subcentral area | 0.52  0.27  0.17  0.02  0.02 |
| CH27 | S11-D10 | 57 13 38 | 44 - pars opercularis, part of Broca's area  6 - Pre-Motor and Supplementary Motor Cortex  9 - Dorsolateral prefrontal cortex | 0.52  0.38  0.10 |
| CH28 | S11-D12 | 53 -1 55 | 6 - Pre-Motor and Supplementary Motor Cortex  4 - Primary Motor Cortex | 0.95  0.05 |
| CH29 | S12-D11 | 62 -47 48 | 40 - Supramarginal gyrus part of Wernicke's area  39 - Angular gyrus, part of Wernicke's area | 0.98  0.02 |
| CH30 | S12-D12 | 55 -31 58 | 1 - Primary Somatosensory Cortex  2 - Primary Somatosensory Cortex  3 - Primary Somatosensory Cortex  40 - Supramarginal gyrus part of Wernicke's area | 0.45  0.11  0.23  0.21 |
| CH31 | S13-D13 | -51 1 54 | 6 - Pre-Motor and Supplementary Motor Cortex  9 - Dorsolateral prefrontal cortex | 0.99  0.01 |
| CH32 | S13-D14 | -54 16 37 | 44 - pars opercularis, part of Broca's area  6 - Pre-Motor and Supplementary Motor Cortex  9 - Dorsolateral prefrontal cortex | 0.75  0.21  0.04 |
| CH33 | S14-D13 | -53 -29 59 | 1 - Primary Somatosensory Cortex  2 - Primary Somatosensory Cortex  3 - Primary Somatosensory Cortex  40 - Supramarginal gyrus part of Wernicke's area | 0.41  0.08  0.40  0.11 |
| CH34 | S14-D15 | -62 -45 47 | 40 - Supramarginal gyrus part of Wernicke's area | 1 |
| CH35 | S15-D13 | -62 -15 45 | 1 - Primary Somatosensory Cortex  2 - Primary Somatosensory Cortex  3 - Primary Somatosensory Cortex  4 - Primary Motor Cortex  43 - Subcentral area | 0.36  0.02  0.35  0.23  0.04 |
| CH36 | S15-D14 | -65 -2 26 | 43 - Subcentral area  6 - Pre-Motor and Supplementary Motor Cortex  4 - Primary Motor Cortex | 0.76  0.21  0.03 |
| CH37 | S15-D15 | -68 -31 32 | 2 - Primary Somatosensory Cortex  40 - Supramarginal gyrus part of Wernicke's area  48 - Retrosubicular area | 0.56  0.29  0.15 |
| CH38 | S15-D16 | -68 -18 11 | 22 - Superior Temporal Gyrus  21 - Middle Temporal gyrus  43 - Subcentral area  48 - Retrosubicular area | 0.85  0.11  0.02  0.02 |
| CH39 | S16-D14 | -61 12 10 | 6 - Pre-Motor and Supplementary Motor Cortex  48 - Retrosubicular area  44 - pars opercularis, part of Broca's area | 0.42  0.38  0.20 |
| CH40 | S16-D16 | -68 -7 -10 | 21 - Middle Temporal gyrus | 1 |

Appendix Table 2: Brodmann area (BA) obtained from MNI coordinates conversion
