# Supplementary material for: Neural responses to social touch with different emotional valences: an fNIRS study
Source: Soc Cogn Affect Neurosci. 2025 Jun 30;20(1):nsaf066. doi: 10.1093/scan/nsaf066 (PMC12380468; doi:10.1093/scan/nsaf066)
Supplement: nsaf066_Supplementary_Data [file nsaf066_supplementary_data.zip › scan-24-277-File014.docx]

**Evaluation of Social Touch Stimuli**

The selection of social touch stimuli was derived from a social touch expression database (Lee Masson and Beeck., 2018). Sixty-two participants evaluated 40 stimuli, and the results were statistically analyzed to calculate the mean and standard deviation of the valence ratings for each stimulus. Ultimately, 16 stimuli were selected, comprising 8 positive stimuli and 8 negative stimuli. The table below presents the information on the 16 stimuli included in the final experiment (See Appendix 1).

|  |
| --- |

| Number | Social Touch Stimuli | M | SD | Valence |
| --- | --- | --- | --- | --- |
| 1 | Handshake | 5.94 | 1.30 | positive |
| 2 | Hug | 5.58 | 1.72 | positive |
| 3 | Shoulder Massage | 6.97 | 1.63 | positive |
| 4 | Close Touch Of Upper Arm | 5.61 | 1.79 | positive |
| 5 | Distant Touch Of Upper Arm | 5.34 | 1.82 | positive |
| 6 | Neck Caress | 5.48 | 1.21 | positive |
| 7 | Hands On Shoulders From Behind | 5.82 | 1.71 | positive |
| 8 | Comforting Hug With Patting | 5.52 | 1.70 | positive |
| 9 | Back Slap | 3.02 | 1.51 | negative |
| 10 | Shoulder Slap | 3.76 | 1.55 | negative |
| 11 | Forearm Slap | 3.89 | 1.61 | negative |
| 12 | Neck Slap From Behind | 4.98 | 1.16 | negative |
| 13 | Shoulder Shake | 4.00 | 1.37 | negative |
| 14 | Forceful Shoulder Tap From Behind | 3.65 | 1.70 | negative |
| 15 | Forceful Elbow Grab | 3.77 | 1.51 | negative |
| 16 | Hand Restraint | 3.79 | 1.57 | negative |

Appendix Table 1: detailed information on the 16 social touch stimuli.

Reference: Lee Masson, H., & Op de Beeck, H. (2018). Socio-affective touch expression database. PloS one, 13(1), e0190921.
